# High bleeding risk and outcomes in left main percutaneous coronary intervention: prognostic value of the PRECISE-DAPT score

**DOI:** 10.1186/s12872-026-05651-w

**Published:** 2026-03-10

**Authors:** En-Shao Liu, Yan-Ning Shih, Yi-Ting Wu, Haw-Ting Tai, Kuo-Ming Yang, Ta-Hsin Tai, Cheng-Hung Chiang, Feng-Yu Kuo

**Affiliations:** 1 Cardiovascular Medical Center, Kaohsiung Veterans General Hospital, No. 386, Dazhong 1st Rd., Zuoying Dist., Kaohsiung City, 813414 Taiwan; 2 Institute of Clinical Medicine, National Yang Ming Chiao Tung University, Taipei, Taiwan; 3 Department of Cardiology, Kaohsiung Municipal United Hospital, Kaohsiung, Taiwan; 4 Department of Pharmacy and Master Program, College of Pharmacy and Health Care, Tajen University, Pingtung, Taiwan

**Keywords:** Left main coronary artery disease, Percutaneous coronary intervention, PRECISE-DAPT score, High bleeding risk

## Abstract

**Objective:**

The PRECISE-DAPT score is a standardized tool for assessing bleeding risk. However, its prognostic value in patients undergoing left main (LM) percutaneous coronary intervention (PCI) remains unclear. This study evaluated the association between high bleeding risk (HBR), defined by the PRECISE-DAPT score, and clinical outcomes following LM PCI.

**Methods:**

This retrospective study analyzed consecutive patients undergoing LM PCI at a tertiary medical center. Patients were stratified into HBR (score ≥ 25) and non-HBR (score < 25) groups. The primary endpoint was major adverse cardiovascular and cerebrovascular events (MACCE), a composite of all-cause death, myocardial infarction, or stroke at one year.

**Results:**

Among 489 patients, 251 (51.3%) were classified as HBR. Compared with the non-HBR group, HBR patients exhibited significantly greater frailty, a higher burden of comorbidities, and more complex lesion characteristics. In multivariate Cox regression analysis, HBR was independently associated with an increased risk of MACCE (adjusted HR: 4.22; 95% CI: 2.03 to 8.77; *P* < 0.001) and bleeding events (adjusted HR: 5.11; 95% CI: 1.46 to 17.90; *P* = 0.01). Harrell’s C-index demonstrated good discrimination for MACCE (0.75; 95% CI: 0.70 to 0.79) and moderate discrimination for bleeding events (0.68; 95% CI: 0.57 to 0.79).

**Conclusion:**

HBR, as determined by the PRECISE-DAPT score, is prevalent among patients undergoing LM PCI and associates with increased frailty, comorbidity burden, and adverse ischemic and bleeding outcomes. The PRECISE-DAPT score effectively stratifies systemic risk and supports its integration into clinical decision-making for this high-risk population.

**Graphical Abstract:**

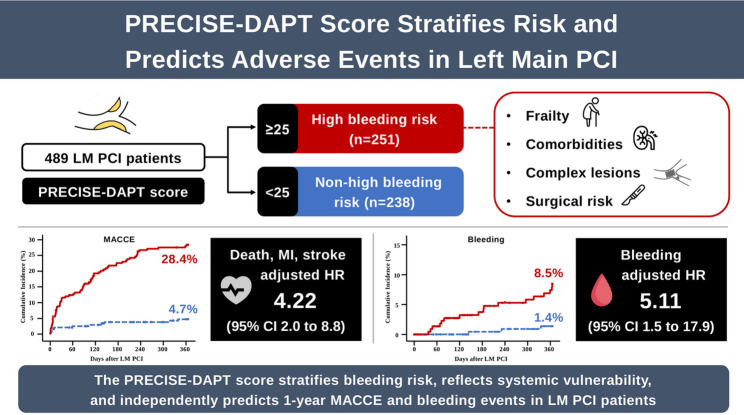

**Supplementary Information:**

The online version contains supplementary material available at 10.1186/s12872-026-05651-w.

## Introduction

Left main (LM) coronary artery disease (CAD) presents significant clinical challenges due to the substantial myocardial territory at risk and elevated potential for adverse cardiovascular outcomes [1]. Although coronary artery bypass grafting (CABG) has long been the standard treatment, advancements in stent technology and interventional techniques have made percutaneous coronary intervention (PCI) a viable alternative in selected patients [2, 3]. This strategy is particularly relevant in Asian populations, where smaller coronary vessels and a cultural reluctance toward CABG often influence revascularization choices [4, 5]. 

Dual antiplatelet therapy (DAPT) is essential after PCI to reduce ischemic events; however, prolonged use increases bleeding risk [6]. [7] Given the high prevalence of comorbidities in LM PCI patients, accurate bleeding risk assessment is critical [8]. The PRECISE-DAPT (PREdicting bleeding Complications In patients undergoing Stent implantation and subsEquent Dual Anti Platelet Therapy) score is a validated, standardized tool for estimating bleeding risk in general PCI populations [9]. Nevertheless, its prognostic performance in LM PCI remains uncertain. This study aimed to explore the impact of high bleeding risk (HBR), as determined by the PRECISE-DAPT score, on clinical outcomes in patients treated with LM PCI.

## Methods

### Study design and patient population

This retrospective study included consecutive patients who underwent their first LM PCI at Kaohsiung Veterans General Hospital, a high-volume tertiary medical center in Taiwan, between January 2010 and September 2023. A high-volume institution was defined as one performing at least 800 PCI procedures annually and providing 24/7 acute coronary syndrome (ACS) care, in accordance with PCI training center guidelines [2]. All patients, or their legal representatives, provided written consent for LM PCI and to forgo CABG, except in cases with prior CABG or hemodynamic instability necessitating urgent revascularization. Consistent with national accreditation standards, the majority of patients with complex coronary anatomy underwent multidisciplinary Heart Team evaluation to validate the revascularization strategy before PCI.

Patients were excluded if they presented with ST-elevation myocardial infarction (STEMI), had previously undergone LM PCI, or lacked post-procedural follow-up in the outpatient department. Those with isolated ostial lesions of the left anterior descending or left circumflex artery were also excluded if the stent did not extend into the LM segment. The date of LM PCI was designated as the index date.

Demographic characteristics, angiographic data, and clinical outcomes were obtained through chart reviews, electronic medical records, and the catheterization laboratory database. All procedures were performed using standard PCI techniques, with treatment strategies determined at the operator’s discretion. DAPT regimen and duration were guided by clinical presentation and physician judgment. The uniformity of clinical documentation was facilitated by Taiwan’s national health insurance system, hospital accreditation standards, and disease-specific care programs for coronary artery disease and acute myocardial infarction (MI), ensuring the quality and consistency of data [10–12]. 

This study did not involve patient or public participation in its design, conduct, or reporting. Ethical approval was granted by the Human Research Committee of Kaohsiung Veterans General Hospital, with informed consent waived due to the retrospective nature of the study.

### Definitions and endpoints

The PRECISE-DAPT score was calculated according to its original algorithm, incorporating five variables: age, hemoglobin, white blood cell count, creatinine clearance, and history of prior bleeding. Scores were determined using the official online calculator (http://www.precisedaptscore.com). Laboratory values were obtained from the most recent results prior to LM PCI. A history of Bleeding Academic Research Consortium (BARC) type 2, 3, or 5 bleeding was identified if any of the following criteria were met: (1) a documented hemorrhagic diagnosis before LM PCI, (2) transfusion for bleeding with associated clinical signs or symptoms, or (3) imaging or endoscopic evidence of bleeding accompanied by symptoms. Patients were stratified into HBR (PRECISE-DAPT score ≥ 25) and non-HBR (score < 25) groups for subsequent analysis.

Anatomical complexity of CAD was assessed using the SYNTAX (Synergy Between PCI with Taxus and Cardiac Surgery) score. Left ventricular ejection fraction (LVEF) was recorded if echocardiography was performed during or within one year before the index hospitalization. Missing LVEF values were imputed using the median of available data to preserve sample size and reduce bias. No imputation was required for other variables. Frailty was assessed using the Clinical Frailty Scale (CFS), which evaluates multimorbidity, function, mobility, and cognition [13, 14]. Surgical risk was estimated using the age, creatinine, and ejection fraction (ACEF) II score, a validated predictor of 30-day cardiac surgery mortality [15, 16]. Detailed definitions of all variables are provided in Supplementary Tables S1 and S2.

The primary outcome was the composite of major adverse cardiac and cerebrovascular events (MACCE) at one-year follow-up, including all-cause death, MI, or stroke. Deaths were categorized as cardiovascular or non-cardiovascular (Supplementary Table S1 and S3), with uncertain causes adjudicated as cardiovascular in origin. Causes of death were ascertained by cross-referencing institutional records with Taiwan’s National Health Insurance Research Database.

Secondary outcomes included the individual components of MACCE, as well as target lesion revascularization (TLR) and target vessel revascularization (TVR). TLR was defined as repeat revascularization within 5 mm proximal or distal to the stent edge, while TVR referred to revascularization of any segment within the target vessel, including its branches. Bleeding outcomes encompassed BARC type 2, 3, or 5 events occurring either during hospitalization or after discharge.

### Statistical analysis

Continuous variables were reported as mean ± standard deviation or median with interquartile range, and compared using Student’s t-test or the Wilcoxon rank-sum test, as appropriate. Categorical variables were expressed as counts and percentages, with group comparisons performed using the Chi-square test or Fisher’s exact test, depending on expected cell frequencies. Time-to-event outcomes were analyzed using the Kaplan–Meier method, with group differences assessed by the log-rank test.

Cox proportional hazards models were used to evaluate associations between HBR and clinical outcomes, with results presented as unadjusted and adjusted hazard ratios (HRs) with 95% confidence intervals (CIs). The proportional hazards assumption was verified by testing for interaction between the covariates and time (time-dependent covariate method), and the assumption was satisfied for the endpoints (Supplementary table S4). Univariate analyses were conducted for all baseline and procedural characteristics, except discharge medications. Variables with a P-value < 0.10 were included in multivariate models. To avoid collinearity, variables directly incorporated in the PRECISE-DAPT score (e.g., age, hemoglobin) were excluded from multivariate analysis. A full list of univariate results is provided in Supplementary Tables S5 and S6.

The predictive performance of the PRECISE-DAPT score for 1-year MACCE and bleeding events was assessed using Harrell’s C-index. Comprehensive sensitivity analyses were conducted to assess the findings. First, to mitigate potential selection bias, we excluded patients with prohibitive surgical risk (ACEF II score > 5) to verify the consistency of outcomes in potential surgical candidates. Second, E-values were calculated to evaluate the potential impact of unmeasured confounding [17]. Finally, Kaplan–Meier estimates for MACCE and bleeding events were extended to 2 and 5 years post-procedure to assess long-term prognostic trends.

All statistical tests were two-sided, with *P* < 0.05 considered statistically significant. Analyses were conducted using SPSS version 22.0 (IBM Corp.) and R version 4.4.1 (R Foundation for Statistical Computing).

## Results

### Baseline characteristics

A total of 544 patients underwent LM PCI at our institution during the study period. After excluding 55 patients due to STEMI, repeat LM PCI, or lack of follow-up data, 489 patients who underwent their first LM PCI were included in the final analysis. Of these, 251 (51.3%) patients were classified as HBR (PRECISE-DAPT score ≥ 25), while 238 (48.7%) were categorized as non-HBR (PRECISE-DAPT score < 25).

Baseline clinical characteristics are presented in Table 1. Compared with the non-HBR group, HBR patients were older, more frequently female, and less likely to be current smokers. They also exhibited greater frailty (46% vs. 8.4%, *P* < 0.001) and had a higher prevalence of comorbidities, including diabetes mellitus (DM), hypertension, chronic kidney disease (CKD), heart failure (HF), and prior cerebrovascular accidents (CVA). Baseline laboratory findings revealed that HBR patients had lower hemoglobin levels, platelet counts, and estimated glomerular filtration rates.

**Table 1:** Baseline data and procedural characteristics

	Total	PRECISE-DAPT < 25	PRECISE-DAPT ≥ 25	*P*-value
	*n* = 489	*n* = 238 (48.7%)	*n* = 251 (51.3%)	
Clinical history				
Age, y	68 (60.0–76.0)	62 (57.0–68.0)	74 (67.0–81.0)	< 0.001
Age ≥ 80 y	87 (17.8)	8 (3.4)	79 (31.5)	< 0.001
Male	387 (79.1)	200 (84.0)	187 (74.5)	0.01
BMI	25.0 (23.0–27.0)	25.5 (23.0–27.0)	25.0 (22.8–27.0)	0.146
Smoker				0.003
Current smoker	102 (20.9)	65 (27.3)	37 (14.7)	
Ex-smoker	131 (26.8)	61 (25.6)	70 (27.9)	
Diabetes mellitus	261 (53.4)	98 (41.2)	163 (64.9)	< 0.001
Hypertension	366 (74.8)	163 (68.5)	203 (80.9)	0.002
Hyperlipidemia	337 (68.9)	194 (81.5)	143 (57.0)	< 0.001
Chronic kidney disease	171 (35.0)	14 (5.9)	157 (62.5)	< 0.001
stage 4, 5, or dialysis	118 (24.1)	1 (0.4)	117 (46.6)	< 0.001
Previous MI	96 (19.6)	48 (20.2)	48 (19.1)	0.77
Previous CABG	61 (12.5)	34 (14.3)	27 (10.8)	0.24
Atrial fibrillation	34 (7.0)	14 (5.9)	20 (8.0)	0.37
Heart failure	138 (28.2)	36 (15.1)	102 (40.6)	< 0.001
PAOD	84 (17.2)	36 (15.1)	48 (19.1)	0.24
Prior CVA	46 (9.4)	13 (5.5)	33 (13.1)	0.004
Bleeding history	82 (16.8)	0 (0.0)	82 (32.7)	< 0.001
Laboratory parameters				
White blood cell, x10^9^/L	7 (5.6–8.7)	7 (5.3–7.9)	7 (5.9–9.8)	< 0.001
Hemoglobin, g/dL	13 (10.7–14.2)	14 (12.8–14.6)	11 (9.7–12.7)	< 0.001
eGFR, ml/min/1.73	61 (33.5–78.0)	76 (65.2–85.6)	35 (9.0-58.2)	< 0.001
Presentation				< 0.001
Stable angina	315 (64.4)	171 (71.8)	144 (57.4)	
Unstable angina	70 (14.3)	37 (15.5)	33 (13.1)	
NSTEMI	104 (21.3)	30 (12.6)	74 (29.5)	
LVEF, %	53 (45.0–60.0)	56 (50.0–60.0)	50 (42.0–58.0)	< 0.001
LVEF ≤ 40%	79 (16.2)	24 (10.1)	55 (21.9)	< 0.001
PRECISE-DAPT score	25 (15.0–43.0)	15 (10.0–19.0)	43 (31.0–57.0)	< 0.001
CFS	3 (3.0–5.0)	3 (2.0–3.0)	4 (3.0–5.0)	< 0.001
CFS ≥ 5	135 (27.7)	20 (8.4)	115 (46.0)	< 0.001
ACEF II score	2 (1.2–4.4)	1 (1.0-1.5)	4 (2.0-5.7)	< 0.001
ACEF II score ≥ 3.5	171 (35.0)	30 (12.6)	141 (56.2)	< 0.001
Angiographic parameters				
Extent of disease				0.008
Isolated LM	9 (1.8)	7 (2.9)	2 (0.8)	
LM and SVD	30 (6.1)	21 (8.8)	9 (3.6)	
LM and DVD	99 (20.2)	53 (22.3)	46 (18.3)	
LM and TVD	351 (71.8)	157 (66.0)	194 (77.3)	
SYNTAX score	28 (20.0–36.0)	27 (18.0–35.0)	29 (22.0–38.0)	0.02
SYNTAX score ≥ 33	178 (36.4)	80 (33.6)	98 (39.0)	0.212
Procedural parameters				
Provisional stenting	407 (83.2)	203 (85.3)	204 (81.3)	0.23
Two-stent technique				0.20
T or TAP stenting	9 (1.8)	3 (1.3)	6 (2.4)	
DK-crush stenting	51 (10.4)	19 (8.0)	32 (12.7)	
Culotte stenting	22 (4.5)	13 (5.5)	9 (3.6)	
Dual arterial access	51 (10.4)	31 (13.0)	20 (8.0)	0.09
Drug-eluting stents	477 (97.5)	234 (98.3)	243 (96.8)	0.282
Total stent length > 60 mm	237 (48.5)	120 (50.4)	117 (46.6)	0.400
Intravascular imaging	292 (59.7)	151 (63.4)	141 (56.2)	0.101
Intravascular ultrasound	271 (55.4)	136 (57.1)	135 (53.8)	0.46
OCT	24 (4.9)	18 (7.6)	6 (2.4)	0.008
Procedure time, min	71 (49.0-103.0)	76 (50.8-104.5)	65 (47.0–99.0)	0.06
Hospitalization days				
Hospital stay, days	4 (2.0–9.0)	3 (2.0–6.0)	7 (3.0–16.0)	< 0.001
Intensive care unit stay, days	3 (2.0–7.0)	2 (2.0–3.0)	5 (2.5–12.0)	< 0.001
Medications at discharge				
Aspirin	481 (98.4)	234 (98.3)	247 (98.4)	1.000
P2Y12 inhibitors	488 (99.8)	238 (100)	250 (99.6)	1.000
Warfarin	5 (1.0)	3 (1.3)	2 (0.8)	0.678
NOAC	13 (2.7)	2 (0.8)	11 (4.4)	0.015
Statin	336 (68.7)	184 (77.3)	152 (60.6)	< 0.001
PPI	195 (39.9)	67 (28.2)	128 (51.0)	< 0.001

Values are n (%) or median (IQR, interquartile range)

*BMI* Body Mass Index, *CABG* coronary artery bypass grafting, *PAOD* peripheral arterial occlusive disease, *CVA* cerebrovascular accident, *eGFR* estimated glomerular filtration rate, *NSTEMI* non-ST-elevation myocardial infarction, *LVEF* left ventricular ejection fraction, *CFS* Clinical Frailty Scale, *ACEF* age, creatinine, and ejection fraction, *LM* left main, *SVD* single vessel disease, *DVD* double vessel disease, *TVD* triple vessel disease, *TAP* T and small protrusion, *DK-crush* double kissing crush, *OCT*: optical coherence tomography, *NOAC* non-vitamin K antagonist oral anticoagulants, *PPI* proton pump inhibitors

Angiographic and procedural characteristics are also summarized in Table 1. Approximately one-third of patients presented with non–ST-elevation ACS (*n* = 174, 35.6%), with non–ST-elevation myocardial infarction (NSTEMI) being more common in the HBR group. HBR patients had lower LVEF, more complex coronary anatomy, higher SYNTAX scores, and more extensive coronary disease. The ACEF II score indicated a markedly higher predicted surgical mortality in the HBR group if CABG had been performed [16]. 

Intravascular imaging was used in over half of the cohort, with no significant difference between groups. Procedural approaches, including bifurcation stenting strategies and use of circulatory support, were comparable between groups. However, HBR patients had significantly longer intensive care unit and total hospital stays, exceeding twice the duration of the non-HBR group.

### Clinical outcomes

**Fig. 1:**
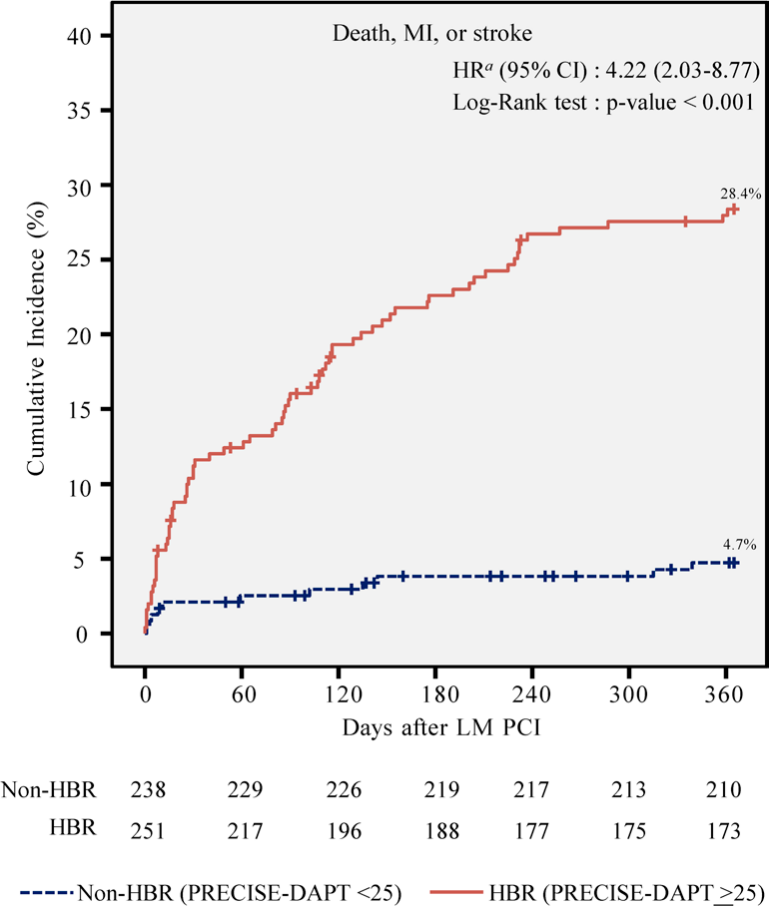
Cumulative 1-year incidence of major adverse cardiovascular and cerebrovascular events (HBR: high bleeding risk, LM: left main, MI: myocardial infarction, PCI: percutaneous coronary intervention)

Among the 489 patients in the analysis, 25 (5.1%) were censored within the first year, and the mean follow-up duration was 321 days. One-year clinical outcomes are summarized in Table 2. The Kaplan-Meier estimated incidence of 1-year MACCE was significantly higher in the HBR group than in the non-HBR group (28.4% vs. 4.7%; log-rank *P* < 0.001; Fig. 1). The HBR group had a 6.86-fold increased hazard of MACCE compared to the non-HBR group (HR: 6.86; 95% CI: 3.63 to 12.95), primarily driven by higher rates of all-cause death (HR: 5.74; 95% CI: 2.92 to 11.25) and MI (HR: 5.86; 95% CI: 1.73 to 19.88) in the HBR group. Cardiovascular mortality was higher in the HBR group (HR: 5.15; 95% CI: 2.29 to 11.59), as was non-cardiovascular mortality (HR: 7.12; 95% CI: 2.11 to 23.95). No significant differences were observed between groups in rates of stroke, TLR, or TVR.

**Table 2:** Clinical outcomes at 1-year follow-up

	Total *n* = 489	PRECISE-DAPT < 25 *n* = 238 (48.7%)	PRECISE-DAPT ≥ 25 *n* = 251 (51.3%)	HR (95% CI)	*P*-value	adjusted HR (95% CI)	*P*-value
Death, MI, or stroke^*^	81 (16.9)	11 (4.7)	70 (28.4)	6.86 (3.63 to 12.95)	< 0.001	4.22 (2.03 to 8.77)	< 0.001
Death	65 (13.3)	10 (4.2)	55 (21.9)	5.74 (2.92 to 11.25)	< 0.001	3.36 (1.53 to 7.36)	0.002
Cardiovascular death	42 (8.7)	7 (3.0)	35 (14.4)	5.15 (2.29 to 11.59)	< 0.001	2.81 (1.08 to 7.33)	0.03
Non-cardiac death	23 (5.0)	3 (1.3)	20 (8.8)	7.12 (2.11 to 23.95)	0.002	4.67 (1.18 to 18.49)	0.03
MI	21 (4.9)	3 (1.3)	18 (8.9)	5.86 (1.73 to 19.88)	0.005	4.24 (1.04 to 17.33)	0.04
Stroke	4 (0.9)	1 (0.4)	3 (1.3)	2.86 (0.30 to 27.46)	0.36	1.13 (0.06 to 21.54)	0.94
TLR	28 (6.7)	6 (2.5)	22 (11.2)	3.58 (1.45 to 8.83)	0.006	1.46 (0.46 to 4.66)	0.52
TVR	36 (8.7)	13 (5.5)	23 (11.7)	1.72 (0.87 to 3.40)	0.12	0.83 (0.33 to 2.11)	0.70
Bleeding events^†^	20 (4.8)	3 (1.3)	17 (8.5)	5.53 (1.62 to 18.88)	0.006	5.11 (1.46 to 17.90)	0.011

Values are presented as n (%). Percentages represent Kaplan-Meier event rates at 1 year following the index procedure

*Model adjusted for diabetes mellitus, hyperlipidemia, chronic kidney disease, heart failure, non-ST-elevation myocardial infarction, multi-vessel disease, left ventricular ejection fraction ≤ 40%

†Bleeding events were defined as Bleeding Academic Research Consortium (BARC) type 2, 3, or 5 bleeding; Model adjusted for hyperlipidemia, previous coronary artery bypass grafting

*MI* myocardial infarction, *TLR* target lesion revascularization, *TVR* target vessel revascularization

**Fig. 2:**
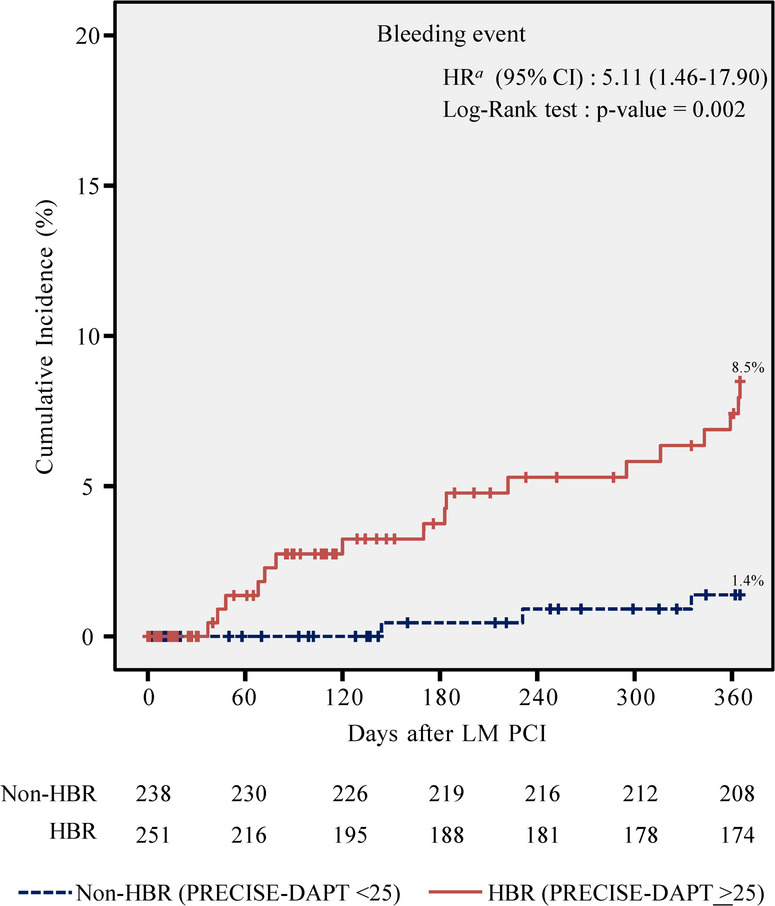
Cumulative 1-year incidence of bleeding events (HBR: high bleeding risk, LM: left main, PCI: percutaneous coronary intervention)

Bleeding events were also significantly more frequent in the HBR group. Kaplan-Meier estimates showed a higher cumulative incidence of bleeding at 1 year in the HBR group compared to the non-HBR group (8.5% vs. 1.4%; log-rank *P* = 0.002; Fig. 2). The risk of bleeding was 5.53 times higher in the HBR group (HR: 5.53; 95% CI: 1.62 to 18.88).

Univariate Cox regression identified several candidate predictors of 1-year MACCE (defined as *P* < 0.10), including a PRECISE-DAPT score ≥ 25, DM, hyperlipidemia, CKD, HF, NSTEMI, LVEF ≤ 40%, and multi-vessel disease (Supplementary Table S5). In multivariate analysis, a PRECISE-DAPT score ≥ 25 remained independently associated with increased MACCE risk (adjusted HR: 4.22; 95% CI: 2.03 to 8.77; *P* < 0.001; Table 3).

**Table 3:** Cox regression analysis for 1-year outcomes in LM PCI

	Univariate HR (95% CI)	*P*-value	adjusted HR (95% CI)	*P*-value
Death, MI, or stroke				
PRECISE-DAPT ≥ 25	6.86 (3.63 to 12.95)	< 0.001	4.22 (2.03 to 8.77)	< 0.001
Diabetes mellitus	1.67 (1.06 to 2.65)	0.03	0.91 (0.55 to 1.49)	0.71
Hyperlipidemia	0.41 (0.27 to 0.64)	< 0.001	0.68 (0.43 to 1.08)	0.10
Chronic kidney disease	3.72 (2.36 to 5.87)	< 0.001	1.53 (0.90 to 2.61)	0.11
Heart failure	1.65 (1.06 to 2.59)	0.03	0.90 (0.56 to 1.44)	0.66
NSTEMI	2.61 (1.67 to 4.09)	< 0.001	1.60 (0.98 to 2.61)	0.06
LVEF ≤ 40%	2.30 (1.42 to 3.72)	0.001	1.44 (0.85 to 2.45)	0.18
Multi-vessel disease^*^	3.67 (0.90 to 14.92)	0.07	2.52 (0.61 to 10.39)	0.20
Bleeding events^†^				
PRECISE-DAPT ≥ 25	5.53 (1.62 to 18.88)	0.006	5.11 (1.46 to 17.90)	0.01
Hyperlipidemia	0.45 (0.19 to 1.07)	0.07	0.58 (0.23 to 1.42)	0.23
Prior CABG	2.40 (0.87 to 6.60)	0.09	2.98 (1.07 to 8.28)	0.04

*Multi-vessel disease: left main with double vessel disease or left main with triple vessel disease

†Bleeding events were defined as Bleeding Academic Research Consortium (BARC) type 2, 3, or 5 bleeding

*LM* left main, *PCI* percutaneous coronary intervention, *NSTEMI* non-ST-elevation myocardial infarction, *CABG* coronary artery bypass grafting

For bleeding events, univariate predictors included a PRECISE-DAPT score ≥ 25, hyperlipidemia, and prior CABG (Supplementary Table S6). Multivariate analysis revealed the PRECISE-DAPT score ≥ 25 as the sole independent predictor of 1-year bleeding events (adjusted HR: 5.11; 95% CI: 1.46 to 17.90; *P* = 0.01; Table 3).

The PRECISE-DAPT score presented good discrimination for 1-year MACCE (Harrell’s C-index = 0.75; 95% CI: 0.70 to 0.79) and moderate discrimination for bleeding (Harrell’s C-index = 0.68; 95% CI: 0.57 to 0.79). In a sensitivity analysis excluding patients with prohibitive surgical risk (ACEF II ≥ 5), a score ≥ 25 remained strongly predictive of both ischemic (HR: 4.70; 95% CI: 2.38 to 9.28; *P* < 0.001) and bleeding events (HR: 5.77; 95% CI: 1.61 to 20.69; *P* = 0.007) (Supplementary Table S7 and Supplementary Figure S1, S2). These findings were robust to unmeasured confounding (E-values: 4.74 for MACCE, 9.69 for bleeding) and sustained through 2-year and 5-year follow-up (Supplementary Figure S3-S6).

## Discussions

This study assessed the prognostic value of HBR, as defined by the PRECISE-DAPT score, in predicting ischemic and bleeding outcomes in patients undergoing LM PCI. The key findings are: (1) HBR is prevalent in this population and associated with greater frailty and comorbidity burden; (2) HBR patients experienced significantly worse outcomes, with the PRECISE-DAPT score serving as a strong independent predictor of 1-year MACCE and bleeding events; and (3) Harrell’s C-index showed good discrimination for MACCE but moderate performance for bleeding, highlighting the complexity of risk prediction in this vulnerable cohort.

### Prevalence of HBR and clinical implications in patients with LM CAD

Our study identified patients with LM CAD as a distinct clinical subset with a high prevalence of HBR, affecting 251 of 489 patients (51.3%). The predominance of elderly patients with multiple comorbidities in the HBR group indicates a strong association between HBR and frailty [18]. Consistent with this, nearly half of the study population demonstrated at least mild frailty based on CFS. Prolonged hospital stays among HBR patients further reflect reduced physiological reserve and slower recovery. Moreover, the complex coronary lesions in this group suggest that HBR reflects not only advanced coronary disease but also a worse overall clinical condition.

Despite the recognized importance of bleeding risk assessment, current evidence remains insufficient to guide revascularization strategies in LM CAD patients with HBR [3]. Our data showed that HBR patients had significantly higher predicted 30-day surgical mortality, with over half having an estimated risk of approximately 10% based on the ACEF II score [16]. This elevated surgical risk may render many HBR patients unsuitable for CABG, potentially explaining why over one-third of patients with high SYNTAX scores (≥ 33) in our cohort underwent LM PCI. However, coexisting multi-vessel disease and long lesion lengths often limit the feasibility of achieving complete revascularization with PCI alone. These findings highlight the complexity of managing LM CAD patients at HBR and underscore the need for further research to develop personalized and effective treatment strategies.

### Impact of PRECISE-DAPT score and HBR on MACCE in LM PCI patients

LM CAD is associated with higher mortality than other forms of CAD [1]. In our study, patients classified as HBR exhibited significantly higher 1-year rates of MACCE. The PRECISE-DAPT score was an independent predictor of MACCE after adjustment for common risk factors (adjusted HR: 4.22; 95% CI: 2.03 to 8.77; *P* < 0.001). The elevated MACCE rate in the HBR group was primarily driven by all-cause mortality and MI. Notably, over one-third of total deaths were from non-cardiac causes, reflecting the high comorbidity burden and age-related vulnerability in this population beyond cardiovascular disease alone.

The PRECISE-DAPT score demonstrated good discriminatory performance for predicting 1-year MACCE (Harrell’s C-index = 0.75; 95% CI: 0.70 to 0.79). HBR was not associated with TLR or TVR, suggesting these outcomes depend more on procedural factors than on baseline characteristics. This underscores the score’s utility in identifying patients at systemic risk, offering prognostic information beyond procedural quality or device-related considerations in LM PCI.

Despite extensive use of intravascular imaging to optimize procedures, the 1-year all-cause mortality in our cohort was higher than reported in other real-world studies [8, 19]. This is likely attributable to the inclusion of a higher-risk population, many of whom were not suitable candidates for CABG due to frailty, comorbidities, or elevated surgical risk. Prior research has reported 6-month mortality rates of 41% in MI patients and 23% in poor CABG candidates undergoing LM PCI, aligning with our findings [20]. Additionally, the long enrollment period in our study may have introduced variability in outcomes, as earlier procedural techniques and devices may have been less advanced than those available in later years [19]. 

### Comparative value of PRECISE-DAPT score versus ARC-HBR criteria

While the Academic Research Consortium for High Bleeding Risk (ARC-HBR) criteria are valuable for prognosis prediction [8], the PRECISE-DAPT score offers distinct advantages in the LM PCI setting. Unlike the binary nature of ARC-HBR, PRECISE-DAPT provides a continuous, weighted risk assessment. Previous studies suggest this approach offers higher specificity for severe bleeding, thereby minimizing false-positive classifications [21–23]. Furthermore, its reliance on objective, universally available laboratory parameters makes it a robust bedside tool, particularly when historical records are incomplete. Notably, our study revealed a potential correlation between PRECISE-DAPT and CFS (Table 1), suggesting that the score may also reflect a patient’s physiological reserve, providing a holistic view of patient vulnerability beyond bleeding risk alone.

### Recognizing HBR and management in patients undergoing LM PCI

Bleeding complications following PCI are well-established predictors of adverse outcomes [24, 25]. Prior studies have shown that patients with HBR derive limited ischemic or mortality benefit from prolonged DAPT, regardless of lesion complexity [6]. Therefore, early identification of HBR is critical to guiding DAPT duration and therapeutic decision-making. In our study, HBR patients undergoing LM PCI had a significantly higher risk of 1-year bleeding events compared to non-HBR patients (adjusted HR: 5.11; 95% CI: 1.46 to 17.90; *P* = 0.01). The PRECISE-DAPT score demonstrated modest discriminatory ability for bleeding (Harrell’s C-index: 0.68; 95% CI: 0.57 to 0.79), consistent with its original validation [9]. 

Although the retrospective design precludes direct comparison of specific regimens, the elevated bleeding risk in patients with PRECISE-DAPT ≥ 25 raises concerns about the safety of a standard DAPT strategy. Consistent with HBR guidelines, clinicians might consider tailored approaches, such as shortened DAPT (1–3 months) followed by P2Y12 inhibitor monotherapy [2, 7]. However, given the catastrophic nature of LM stent thrombosis, such decisions mandate strictly individualized balancing of predicted bleeding risk against anatomical complexity.

This balance is particularly challenging because the PRECISE-DAPT score is capped at 36, which may plateau risk estimation in extreme cases, and clinical variables such as hemoglobin and creatinine clearance often fluctuate over time [9]. Furthermore, the overall fragility of these patients means that even aspirin monotherapy may carry substantial bleeding risk [26, 27]. Managing this complexity requires a comprehensive approach. Beyond optimizing procedures and antithrombotic therapy, clinicians should actively manage anemia and preserve renal function. These measures may help reduce bleeding risk while maintaining adequate ischemic protection. Ultimately, a personalized, holistic management approach that extends beyond procedural factors is essential for improving outcomes in LM CAD patients with HBR.

### Study limitations

Several limitations should be considered when interpreting the findings of this study. First, the retrospective nature of this study is inherently subject to potential unmeasured confounding and selection bias despite statistical adjustments. Key procedural metrics, such as the completeness of revascularization and residual SYNTAX scores, were not assessed. Additionally, detailed information regarding the exact duration, specific regimens, and patient adherence to antithrombotic therapy was unavailable. While DAPT was typically prescribed for at least 6 months, post-discharge modifications—including regimen switching or premature discontinuation—precluded a detailed longitudinal analysis. As pharmacological management drives prognosis, the lack of granular data on compliance and regimen adjustments introduces bias. Uncaptured variations in antithrombotic intensity might also confound the observed association between baseline characteristics and outcomes. Although Heart Team evaluation was standard, specific reasons for declining CABG were not systematically recorded. This limitation may enrich the cohort for frailty and unmeasured comorbidities, potentially inflating event rates relative to randomized trials. Furthermore, the incidence of bleeding, particularly BARC type 2 events, may be underestimated. These events often prompt clinically significant actions, such as therapy withdrawal, but frequently occur in outpatient settings without hospitalization, potentially leading to underreporting.

Second, the study was conducted at a single tertiary medical center where clinical practices may not reflect those of other institutions. Although care followed contemporary standards, differences in institutional protocols or regional practices may limit the generalizability of the findings. Multicenter or prospective studies are warranted to validate the applicability of the PRECISE-DAPT score across diverse populations and clinical settings.

Third, the relatively small sample size limited the power to detect differences in less frequent outcomes, such as stroke, and to evaluate the specific impact of intravascular imaging. Bleeding prediction showed moderate discrimination with wide confidence intervals, likely reflecting low event rates and competing risks in frail LM PCI patients. This underscores that risk scores stratify risk rather than deterministically predict individual bleeding outcomes. Additionally, follow-up at five years was incomplete, rendering long-term outcomes exploratory. Routine follow-up angiography was not performed, which may have affected the assessment of vessel patency.

Despite these limitations, this study is the first to apply the PRECISE-DAPT score to evaluate the impact of high bleeding risk on outcomes in patients undergoing LM PCI. The sensitivity analysis supported the robustness of our findings. The E-values for MACCE (4.74) andbleeding events (9.69) suggest that strong unmeasured confounders would be required to fully explain the observed associations. Moreover, extended follow-up showed persistent separation of Kaplan-Meier event curves through 2 and 5 years, reinforcing the durability and consistency of the identified risk relationships over time.

## Conclusion

The PRECISE-DAPT score demonstrates reliable discriminatory performance for both ischemic and bleeding risk in patients undergoing LM PCI. HBR is common in this population and is closely associated with frailty, substantial comorbidity burden, complex coronary anatomy, and elevated surgical risk. Despite frequent use of intravascular imaging, HBR patients remain at increased risk for MACCE and bleeding. These findings highlight the critical role of systemic, patient-level factors in driving adverse outcomes, beyond procedural complexity alone, and support the PRECISE-DAPT score as a simple, clinically useful tool for risk stratification and guiding future research in this high-risk group.

## Supplementary Information

Supplementary Material 1: Supplementary Table S1. Study definitions. Supplementary Table S2. Comparison of BARC bleeding and study definition. Supplementary Table S3. Causes of non-cardiac deaths. Supplementary Table S4. Test of proportional hazards assumption. Supplementary Table S5. Univariate Cox regression for 1-year MACCE in LM PCI. Supplementary Table S6. Univariate Cox regression for 1-year bleeding events in LM PCI. Supplementary Table S7. Clinical outcomes at 1-year follow-up in patients with ACEF II score <5. Supplementary Figure S1. Cumulative 1-year incidence of major adverse cardiovascular and cerebrovascular events in patients with ACEF II Score <5. Supplementary Figure S2. Cumulative 1-year incidence of bleeding events in patients with ACEF II Score <5. Supplementary Figure S3. Cumulative two-year incidence of major adverse cardiovascular and cerebrovascular events. Supplementary Figure S4. Cumulative two-year incidence of bleeding events. Supplementary Figure S5. Cumulative five-year incidence of major adverse cardiovascular and cerebrovascular events. Supplementary Figure S6. Cumulative five-year incidence of bleeding events.

## Data Availability

The datasets generated and analyzed during the current study are not publicly available due to institutional review board (IRB) policies and restrictions under Taiwan’s Personal Data Protection Act. De-identified data may be made available upon reasonable request, subject to a data-use agreement and IRB approval, and only within the scope permitted by applicable regulations. Requests for access to the data should be directed to the corresponding author, Feng-Yu Kuo (E-mail: fykuocv@gmail.com).

## Abbreviations

ACS: acute coronary syndrome
ACEF: age, creatinine, and ejection fraction
ARC-HBR: Academic Research Consortium for High Bleeding Risk
BARC: Bleeding Academic Research Consortium
CABG: coronary artery bypass grafting
CAD: coronary artery disease
CI: confidence interval
CKD: chronic kidney disease
CFS: Clinical Frailty Scale
CVA: cerebrovascular accidents
DAPT: dual antiplatelet therapy
DM: diabetes mellitus
LVEF: left ventricular ejection fraction
HBR: high bleeding risk
HF: heart failure
HR: hazard ratio
LM: left main
MACCE: major adverse cardiac and cerebrovascular events
MI: myocardial infarction
NSTEMI: non-ST elevation myocardial infarction
PCI: percutaneous coronary intervention
PRECISE-DAPT: PREdicting bleeding Complications In patients undergoing Stent implantation and subsEquent Dual Anti Platelet Therapy
STEMI: ST-elevation myocardial infarction
SYNTAX: synergy between percutaneous coronary intervention with taxus and cardiac surgery
TLR: target lesion failure
TVR: target vessel failure
