# Supplementary material for: High bleeding risk and outcomes in left main percutaneous coronary intervention: prognostic value of the PRECISE-DAPT score
Source: BMC Cardiovasc Disord. 2026 Mar 10;26:324. doi: 10.1186/s12872-026-05651-w (PMC13085693; doi:10.1186/s12872-026-05651-w)
Supplement: Supplementary file 1 — Supplementary Material 1: Supplementary Table S1. Study definitions. Supplementary Table S2. Comparison of BARC bleeding and study definition. Supplementary Table S3. Causes of non-cardiac deaths. Supplementary Table S4. Test of proportional hazards assumption. Supplementary Table S5. Univariate Cox regression for 1-year MACCE in LM PCI. Supplementary Table S6. Univariate Cox regression for 1-year bleeding events in LM PCI. Supplementary Table S7. Clinical outcomes at 1-year follow-up in patients with ACEF II score <5. Supplementary Figure S1. Cumulative 1-year incidence of major adverse cardiovascular and cerebrovascular events in patients with ACEF II Score <5. Supplementary Figure S2. Cumulative 1-year incidence of bleeding events in patients with ACEF II Score <5. Supplementary Figure S3. Cumulative two-year incidence of major adverse cardiovascular and cerebrovascular events. Supplementary Figure S4. Cumulative two-year incidence of bleeding events. Supplementary Figure S5. Cumulative five-year incidence of major adverse cardiovascular and cerebrovascular events. Supplementary Figure S6. Cumulative five-year incidence of bleeding events. [file 12872_2026_5651_MOESM1_ESM.docx]

**Supplementary Table S1. Study definitions**

|  | Definition used in study |
| --- | --- |
| Baseline characteristics |  |
| Chronic kidney disease | Defined as an eGFR <60 mL/min/1.73 m² or a documented diagnosis of CKD   - CKD stage 3: eGFR 30-59 mL/min/1.73 m² - CKD stage 4, 5, or dialysis: eGFR <30 mL/min/1.73 m² or regular dialysis |
| PRECISE-DAPT score | Calculated using an online tool (http://www.precisedaptscore.com) based on age, Hgb, WBC, creatinine clearance, and prior bleeding history. Values were taken from the last available data before LM PCI. A score ≥25 indicates high bleeding risk (HBR). |
| Clinical Frailty Scale | The degree of frailty was assessed using the 9-point Clinical Frailty Scale (CFS). Frailty classification was conducted through chart review by both a physician and a research assistant. In cases where there was a discrepancy in the assigned CFS score, the final determination was made by an independent head nurse who was not involved in the research process. A score ≥5 indicates at least mild frailty. |
| Laboratory parameters | |
| WBC, Hgb, Hct, platelet, and Cr | The last available laboratory results prior to LM PCI during the index admission. No missing data were reported for these variables. |
| eGFR, ml/min/1.73 | Calculated using the Modification of Diet in Renal Disease (MDRD) equation.  No missing data were reported. |
| LVEF, % | Documented from the closest available echocardiography performed during or within 1 year prior to the index admission for LM PCI. Data were unavailable for 21 patients. The missing data was handled with median imputation. A value ≤40% indicates reduced ejection fraction. |
| ACEF II score | ACEF II= Age/EF + 2.0 (if serum creatinine >2.0mg/dL) + 3.0 (if emergency surgery) + 0.2 x hematocrit (points below 36%)   - The age and documented LVEF values used were within a 1-year timeframe - Emergency surgery was defined as patients presenting with non ST elevation myocardial infraction - Hematocrit values of 36% or higher count as zero - A score ≥3.5 indicates a predicted 30-day surgical mortality risk of approximately 10%. |
| Procedural parameters |  |
| Dual arterial access | Use of two arterial access (e.g., distal radial, radial, brachial, or femoral access) concurrently during the procedure. |
| Intravascular imaging |  |
| IVUS or OCT | Use of intravascular imaging devices to assess the LM lesion or ostial lesions of the left anterior descending or left circumflex artery |
| Outcomes |  |
| Cardiovascular death | Defined by the principal diagnosis listed in medical records for death. Causes included myocardial infarction, heart failure, sudden cardiac death, ventricular arrhythmia, stroke, or procedural complications (e.g., PCI-related). Deaths with no clear attributable cause were classified as cardiac in origin. |
| Non-cardiac death | Defined based on the principal diagnosis listed in medical records, attributed to clearly identifiable non-cardiac conditions such as cancer, septic shock, or other non-cardiovascular causes. |
|  | Mortality data were obtained by linking patient records to Taiwan’s National Health Insurance Research Database to capture out-of-hospital deaths. This was cross-referenced with institutional electronic medical records.   - For in-hospital deaths, the cause was determined based on the discharge diagnosis and death certificate in the medical records. - For out-of-hospital deaths, the principal diagnosis recorded in the National Health Insurance Research Database at the time of death was used. - A designated physician first reviewed the records to determine the cause of death. A second physician then reviewed this determination. The corresponding author addressed any discrepancies between the two reviewers. All assessors were not blinded to patient characteristics due to the need for comprehensive medical record review. |

LM: left main, PCI: percutaneous coronary intervention, CKD: chronic kidney disease, WBC: white blood cell, Hgb: hemoglobin, Hct: hematocrit, eGFR: estimated glomerular filtration rate, LVEF: left ventricular ejection fraction, IVUS: intravascular ultrasound, OCT: optical coherence tomography

**Supplementary Table S2. Comparison of BARC bleeding and study definition**

| Bleeding Academic Research Consortium (BARC) bleeding | - BARC 2: Overt bleeding requiring non-surgical medical care or resulting in hospitalization. - BARC 3: Drop of >3 g/dL in Hgb, any transfusion, cardiac tamponade, intra-cranial hemorrhage, intra-ocular bleeding with vision compromise, or bleeding requiring surgical intervention or vasopressors - BARC 5: Fatal bleeding |
| --- | --- |
| Study operational definition | Unlike the Academic Research Consortium-High Bleeding Risk (ARC-HBR) criteria, which defines bleeding events within the past 12 months, the PRECISE-DAPT score does not specify a time frame for bleeding history. To account for patients' potentially extensive medical histories before undergoing left main percutaneous coronary intervention, we used the following methods in electronic medical records for identifying bleeding histories in our institution:   1. Hemorrhage-related diagnosis: Documented in the outpatient department, emergency room, or during prior hospitalization using specific ICD codes. 2. Blood transfusion codes: Administered in the presence of clinical signs or symptoms of bleeding. 3. Endoscopic or imaging evidence: Bleeding confirmed through endoscopy or imaging studies, accompanied by clinical symptoms or signs.    - Examination codes: Included codes such as ultrasound, slit-lamp examinations, computed tomography, and magnetic resonance imaging.    - Procedure Codes: Included esophagogastroduodenoscopy (EGD), colonoscopy, bronchoscopy, and cystoscopy.   In cases where bleeding events did not meet BARC criteria but were identified in our institutional database, additional chart reviews were conducted to confirm their validity. E.g., Patients with chronic kidney disease who received blood transfusions solely for renal anemia without evidence of bleeding were not recorded as having a bleeding event. |
| Central nervous system hemorrhage | ICD-9: 430, 431, 432.0, 432.1, 432.9, 852.2, 852.4, 852.5, 998.1  ICD-10: I60.0, I60.1, I60.2, I60.3, I60.4, I60.5, I60.6, I60.7, I60.8, I60.9, I61.0, I61.1, I61.2, I61.3, I61.4, I61.5, I61.6, I61.8, I61.9, I62.0, I62.1, I62.9, S06.4, S06.5, S06.6 |
| Gastrointestinal hemorrhage | ICD-9: 530.7, 531.0, 532.0, 533.0, 534.0, 535.01, 569.3, 578.0, 578.1, 578.9  ICD-10: K22.6, K25.0, K25.2, K25.4, K25.6, K26.0, K26.2, K26.4, K26.6, K27.0, K27.2, K27.4, K27.6, K28.0, K28.2, K28.4, K28.6, K29.0, K62.5, K92.0, K92.1, K92.2 |
| Genitourinary hemorrhage | ICD-9: 599.7, 626.2, 626.6, 626.8, 626.9, 627.1  ICD-10: N02.0, N02.1, N02.2, N02.3, N02.4, N02.5, N02.6, N02.7, N02.8, N02.9, N42.1, N92.0, N92.1, N92.3, N92.4, N93.0, N93.8, N93.9, R31, R04.1 |
| Other hemorrhage | ICD-9: 786.3, 786.39, 362.81, 379.23, 423.0, 372.72, 719.1, 568.81, 861.1, 860.0, 285.1, 286.5, 287.8, 287.9, 459.0, 784.7, 784.8  **ICD-10**: R04.2, R04.8, J94.2, H35.6, H43.1, H31.3, I31.2, H11.3, M25.0, K66.1, S26.0, S27.1, T79.2, D62, D68.3, D69.8, D69.9, R58, R04.0, R04.9 |

**Supplementary Table S3. Causes of non-cardiovascular deaths**

| Age/sex | PRECISE DAPT score | Time from LM PCI to event | Medical history | Cause of non-cardiovascular death |
| --- | --- | --- | --- | --- |
| 83 male | 25 | 27 days | DM, PAOD | Aspiration pneumonia with respiratory failure |
| 88 male | 57 | 204 days | DM, HTN, hyperlipidemia, CKD, PAOD | Accidental injury death |
| 83 male | 50 | 61 days | COPD | COPD with emphysematous change and multifocal pneumonia with respiratory failure |
| 60 female | 46 | 115 days | DM, HTN, CKD | Candida rugosa fungemia, catheter related blood stream infection related, with septic shock |
| 78 female | 65 | 361 days | DM, CKD, GI bleeding | Perineal abscess with Enterococcus faecalis bacteremia and septic shock |
| 90 female | 37 | 201 days | DM, HTN, hyperlipidemia, prior CABG, HF, rectal cancer | Rectal adenocarcinoma, s/p concurrent chemoradiotherapy, with ulcerative radiation proctitis and refractory bleeding |
| 73 female | 80 | 107 days | DM, HTN, hyperlipidemia, CKD, HF, GI bleeding | Proteus mirabilis bacteremia, infection focus undetermined, with septic shock |
| 70 male | 73 | 147 days | DM, hyperlipidemia, CKD, prior MI, HF, hematuria | Methicillin resistant Staphylococcus aureus (MRSA) bacteremia, Permcath catheter related, with septic shock |
| 71 male | 18 | 315 days | DM, HTN, lymphoma | Diffuse large B cell lymphomas with central nervous system involvement, stage IV |
| 63 male | 35 | 191 days | DM, hyperlipidemia, GI bleeding, cirrhosis, hepatocellular carcinoma | Decompensated cirrhosis with esophageal varices bleeding, hypovolemic shock and multi-organ failure |
| 39 male | 7 | 3 days | DM, HTN, hyperlipidemia, prior CABG | Right-sided multiple rib fractures and traumatic hemothorax secondary to traffic accident |
| 89 male | 80 | 25 days | HTN, CKD, prior MI, PAOD, HF, COPD, GI bleeding | Pneumonia of bilateral lungs, Pseudomonas aeruginosa related, with septic shock and respiratory failure |
| 53 male | 39 | 129 days | DM, HTN, CKD, prior MI, PAOD, nasopharyngeal cancer | Osteomyelitis of right tibia and metatarsal bone, Carbapenem-resistant Acinetobacter baumannii (CRAB) related, with sepsis and acidosis |
| 90 female | 58 | 15 days | HTN, CKD, Af, prior CVA | Fall injury with traumatic Type II Odontoid fracture and posterior displacement, with retropharyngeal hematoma and respiratory failure. |
| 82 female | 37 | 287 days | DM, HTN, pancreas cancer | Pancreas cancer, cT3N1M1, with peritoneal carcinomatosis |
| 83 male | 68 | 7 days | DM, CKD, PAOD, HF | Hospital-acquired pneumonia, with respiratory failure |
| 64 male | 67 | 85 days | DM, HTN, CKD, PAOD, GI bleeding | Trans-tibial amputation stump infection with Citrobacter koseri bacteremia and septic shock |
| 85 male | 36 | 30 days | DM, prior MI | Incarcerated small bowel herniation into left inguinal canal, with bowel obstruction and secondary intra-abdominal infection |
| 69 female | 69 | 14 days | DM, HTN, CKD, PAOD, Af, GI bleeding | Refractory ventilator-associated pneumonia, Klebsiella pneumoniae and methicillin-resistant Staphylococcus aureus (MRSA) related, with septic shock |
| 66 male | 11 | 135 days | HTN, hyperlipidemia, prior CABG, lung cancer | Adenocarcinoma of left lung, with brain and pons metastases, cT2N3M1b, stage IVb |
| 74 female | 45 | 31 days | DM, hyperlipidemia, CKD | Closed-loop small bowel obstruction secondary to adhesive band, complicated with mesenteric congestion and intra-abdominal infection |
| 66 male | 39 | 5 days | DM, HTN, hyperlipidemia, CKD | Acute kidney injury, suspected medications, dehydration, and contrast related, with hyperkalemia |
| 76 male | 55 | 86 days | HTN, CKD, HF | Acute hepatitis, suspected herbal medicine related, with hepatic failure and hepatic encephalopathy |

Af: atrial fibrillation, CABG: coronary artery bypass grafting, CKD: chronic kidney disease, CVA: cerebrovascular accident, DM: diabetes mellitus, GI: gastrointestinal, HF: heart failure, HTN: hypertension, MI: myocardial infarction, PAOD: peripheral arterial occlusive disease,

**Supplementary Table S4. Test of proportional hazards assumption**

| **Outcome** | Variable tested | Interaction with time (coefficient) | P-value for interaction |
| --- | --- | --- | --- |
| MACCE | PRECISE-DAPT ≥25 vs <25 | 0.000 | 0.957 |
| Bleeding event | PRECISE-DAPT ≥25 vs <25 | -0.003 | 0.594 |

The proportional hazards assumption was tested by including a time-dependent covariate (interaction of the predictor with time) in the Cox model. A non-significant P-value indicates that the assumption satisfies

**Supplementary Table S5. Univariate Cox regression for 1-year MACCE in LM PCI**

|  | Univariate HR (95% CI) | P-value |
| --- | --- | --- |
| Clinical history |  |  |
| Age^§^ | 1.04 (1.02 to 1.07) | <0.001 |
| Female | 1.36 (0.83 to 2.23) | 0.23 |
| Smoking history |  |  |
| Non-smoker | Ref |  |
| Current smoker | 0.82 (0.47 to 1.45) | 0.50 |
| Ex-smoker | 0.67 (0.38 to 1.16) | 0.15 |
| **Diabetes mellitus** | **1.67 (1.06 to 2.65)** | **0.03** |
| Hypertension | 1.17 (0.69 to 1.98) | 0.55 |
| **Hyperlipidemia** | **0.41 (0.27 to 0.64)** | **<0.001** |
| **Chronic kidney disease** | **3.72 (2.36 to 5.87)** | **<0.001** |
| Previous MI | 0.61 (0.32 to 1.15) | 0.13 |
| Prior CABG | 1.09 (0.58 to 2.05) | 0.80 |
| Atrial fibrillation | 0.68 (0.25 to 1.86) | 0.46 |
| **Heart failure** | **1.65 (1.06 to 2.59)** | **0.03** |
| PAOD | 1.31 (0.77 to 2.23) | 0.33 |
| Prior CVA | 1.41 (0.73 to 2.73) | 0.31 |
| Bleeding history^§^ | 2.13 (1.31 to 3.45) | 0.002 |
| Laboratory parameters |  |  |
| White blood cell, 10^9/L^§^ | 1.16 (1.11 to 1.21) | <0.001 |
| Hemoglobin, g/dL^§^ | 0.73 (0.66 to 0.81) | <0.001 |
| Platelet, 10^9/L | 1.00 (1.00 to 1.00) | 0.71 |
| eGFR, ml/min/1.73^§^ | 0.98 (0.97 to 0.98) | <0.001 |
| Presentations |  |  |
| Non-MI | Ref |  |
| **NSTEMI** | **2.61 (1.67 to 4.09)** | **<0.001** |
| **LVEF ≤40%** | **2.30 (1.42 to 3.72)** | **0.001** |
| **PRECISE-DAPT ≥25** | **6.86 (3.63 to 12.95)** | **<0.001** |
| Angiographic parameters |  |  |
| Extent of CAD |  |  |
| Isolated LM or SVD | Ref |  |
| **Multi-vessel disease**^*^ | **3.67 (0.90 to 14.92)** | **0.07** |
| SYNTAX score | 1.01 (1.00 to 1.03) | 0.16 |
| Procedural parameters |  |  |
| Bifurcation stenting strategy |  |  |
| Provisional stenting | Ref |  |
| Two-stent technique^†^ | 0.67 (0.35 to 1.30) | 0.24 |
| Dual arterial access | 1.29 (0.67 to 2.50) | 0.45 |
| Drug-eluting stents | 2.13 (0.30 to 15.31) | 0.45 |
| Total stent length >60 mm | 0.72 (0.46 to 1.12) | 0.14 |
| Intravascular imaging^‡^ | 0.71 (0.46 to 1.10) | 0.13 |

*Multi-vessel disease: left main with double vessel disease or left main with triple vessel disease

†Two-stent technique: T stenting, T and small protrusion stenting, double kissing crush stenting, or Culotte stenting

‡Intravascular imaging: use of intravascular ultrasound or optical coherence tomography

§Age, bleeding history, white blood cell, hemoglobin, and eGFR are variables of PRECISE-DAPT formula

MACCE: major adverse cardiac and cerebrovascular events, MI: myocardial infarction, PCI: percutaneous coronary intervention, CABG: coronary artery bypass grafting, PAOD: peripheral arterial occlusive disease, CVA: cerebrovascular accident, eGFR: estimated glomerular filtration rate, NSTEMI: non-ST-elevation myocardial infarction, LVEF: left ventricular ejection fraction, CAD: coronary artery disease, LM: left main, SVD: single vessel disease

**Supplementary Table S6. Univariate Cox regression for 1-year bleeding events in LM PCI**

|  | Univariate HR (95% CI) | P-value |
| --- | --- | --- |
| Clinical history |  |  |
| Age^§^ | 1.01 (0.98 to 1.05) | 0.50 |
| Female | 1.26 (0.46 to 3.47) | 0.65 |
| Smoking history |  |  |
| Non-smoker | Ref |  |
| Current smoker | 1.01 (0.32 to 3.21) | 0.99 |
| Ex-smoker | 1.18 (0.43 to 3.25) | 0.75 |
| Diabetes mellitus | 0.71 (0.30 to 1.72) | 0.46 |
| Hypertension | 1.00 (0.36 to 2.76) | 1.00 |
| **Hyperlipidemia** | **0.44 (0.19 to 1.07)** | **0.07** |
| Chronic kidney disease | 1.88 (0.78 to 4.53) | 0.16 |
| Previous MI | 1.03 (0.34 to 3.08) | 0.96 |
| **Prior CABG** | **2.40 (0.87 to 6.60)** | **0.09** |
| PAOD | 2.13 (0.82 to 5.53) | 0.12 |
| Atrial fibrillation | 1.48 (0.34 to 6.40) | 0.60 |
| Heart failure | 1.70 (0.70 to 4.17) | 0.24 |
| Prior CVA | 1.07 (0.25 to 4.62) | 0.93 |
| Bleeding history^§^ | 4.24 (1.76 to 10.24) | 0.001 |
| Laboratory parameters |  |  |
| White blood cell, 10^9/L^§^ | 0.85 (0.69 to 1.05) | 0.13 |
| Hemoglobin, g/dL^§^ | 0.83 (0.68 to 1.02) | 0.08 |
| Platelet, 10^9/L | 0.99 (0.99 to 1.00) | 0.12 |
| eGFR, ml/min/1.73^§^ | 0.99 (0.98 to 1.01) | 0.31 |
| Presentations |  |  |
| Non-MI | Ref |  |
| NSTEMI | 0.93 (0.31 to 2.78) | 0.90 |
| LVEF ≤40% | 1.31 (0.44 to 3.93) | 0.63 |
| **PRECISE-DAPT ≥25** | **5.53 (1.62 to 18.88)** | **0.006** |
| Angiographic parameters |  |  |
| Extent of CAD |  |  |
| Isolated LM or SVD | Ref |  |
| Multi-vessel disease^*^ | 0.49 (0.14 to 1.67) | 0.25 |
| SYNTAX score | 0.99 (0.95 to 1.03) | 0.54 |
| Procedural parameters |  |  |
| Bifurcation stenting strategy |  |  |
| Provisional stenting | Ref |  |
| Two-stent technique^†^ | 0.55 (0.13 to 2.35) | 0.42 |
| Dual arterial access | 1.54 (0.45 to 5.27) | 0.49 |
| Drug-eluting stents | 0.45 (0.06 to 3.35) | 0.43 |
| Total stent length >60 mm | 0.45 (0.17 to 1.18) | 0.10 |
| Intravascular imaging^‡^ | 0.55 (0.23 to 1.32) | 0.18 |

*Multi-vessel disease: left main with double vessel disease or left main with triple vessel disease

†Two-stent technique: T stenting, T and small protrusion stenting, double kissing crush stenting, or Culotte stenting

‡Intravascular imaging: use of intravascular ultrasound or optical coherence tomography

§Age, bleeding history, white blood cell, hemoglobin, and eGFR are variables of PRECISE-DAPT formula

MI: myocardial infarction, PCI: percutaneous coronary intervention, CABG: coronary artery bypass grafting, PAOD: peripheral arterial occlusive disease, CVA: cerebrovascular accident, eGFR: estimated glomerular filtration rate, NSTEMI: non-ST-elevation myocardial infarction, LVEF: left ventricular ejection fraction, CAD: coronary artery disease, LM: left main, SVD: single vessel disease

**Supplementary Table S7. Clinical outcomes at 1-year follow-up in patients with ACEF II score <5**

|  | Total  n=400 | PRECISE-DAPT <25  n=234 | PRECISE-DAPT ≥25  n=166 | HR  (95% CI) | P-value |
| --- | --- | --- | --- | --- | --- |
| Death, MI, or stroke | 45 (11.5) | 11 (4.8) | 34 (20.9) | 4.70 (2.38-9.28) | <0.001 |
| Bleeding events | 14 (3.9) | 3 (1.4) | 11 (7.8) | 5.77 (1.61-20.69) | 0.007 |

Values are presented as n (%). Percentages represent Kaplan-Meier event rates at 1 year following the index procedure.

MI: myocardial infarction

**Supplementary Figure S1.** **Cumulative 1-year incidence of major adverse cardiovascular and cerebrovascular events in Patients with ACEF II Score <5**


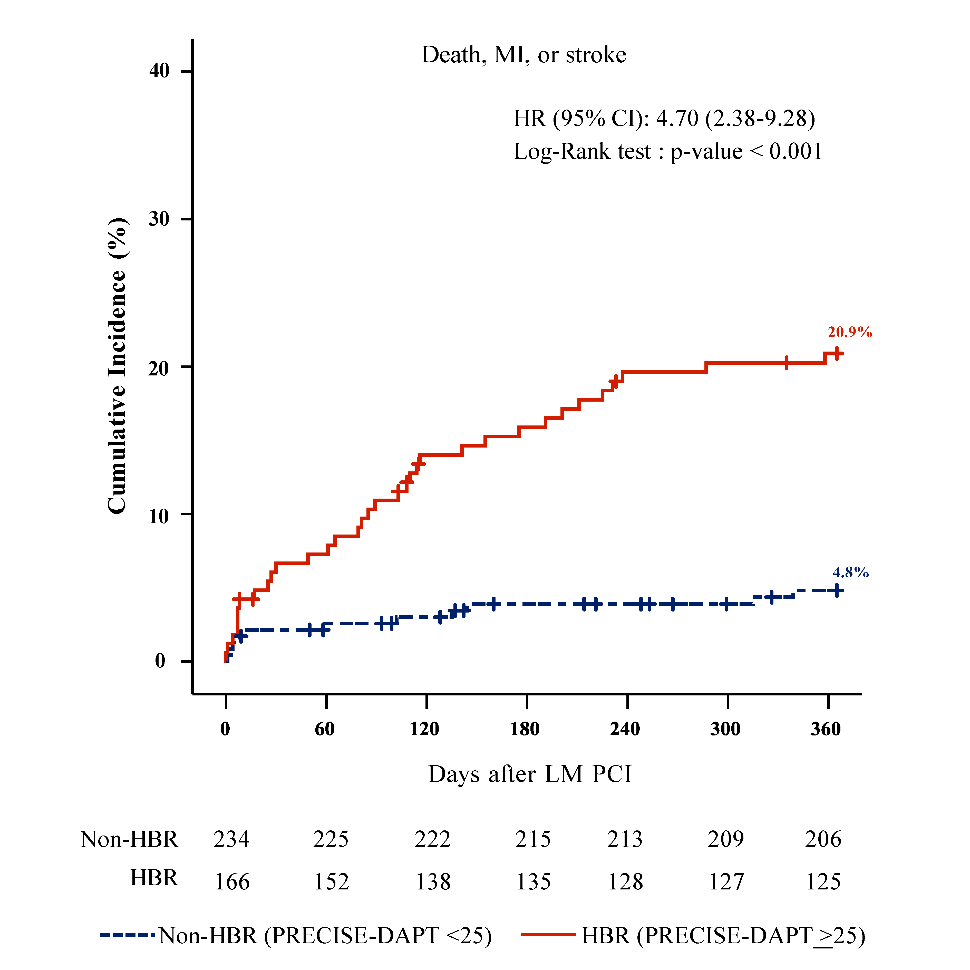


**Supplementary Figure S2.** **Cumulative 1-year incidence of bleeding events in patients with ACEF II Score <5**


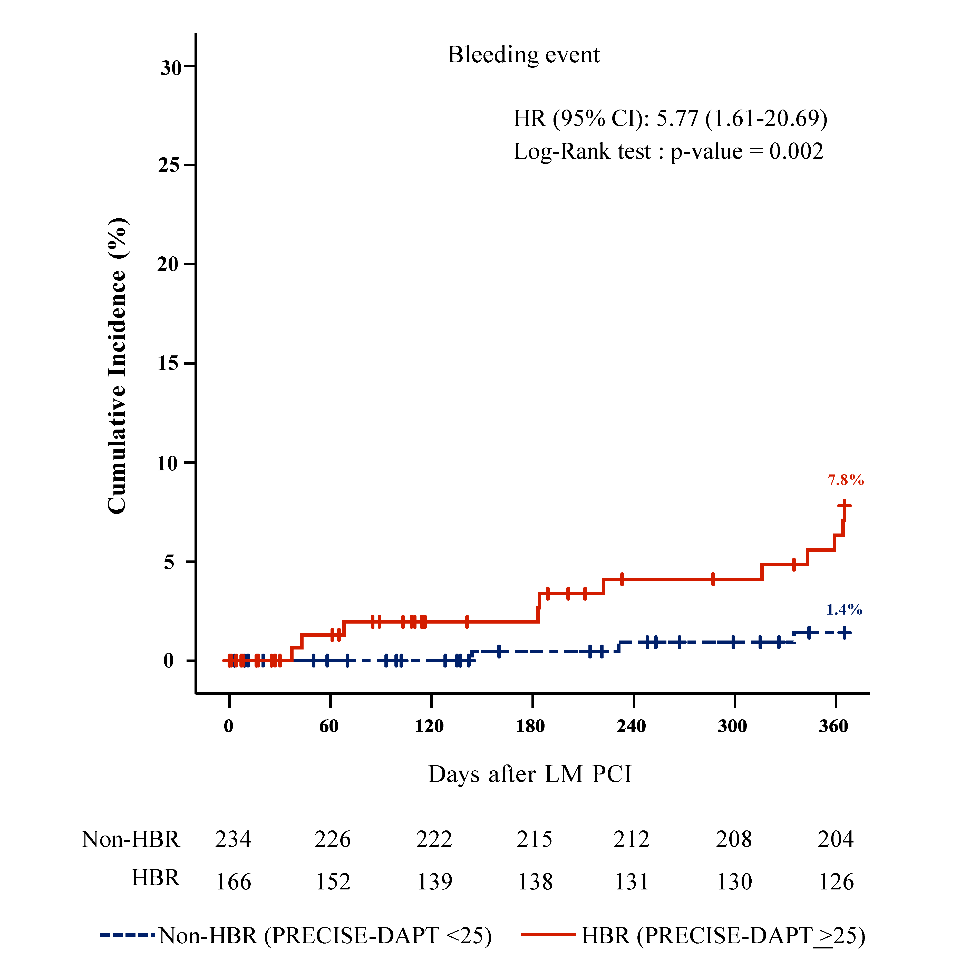


**Supplementary Figure S3. Cumulative two-year incidence of major adverse cardiovascular and cerebrovascular events**


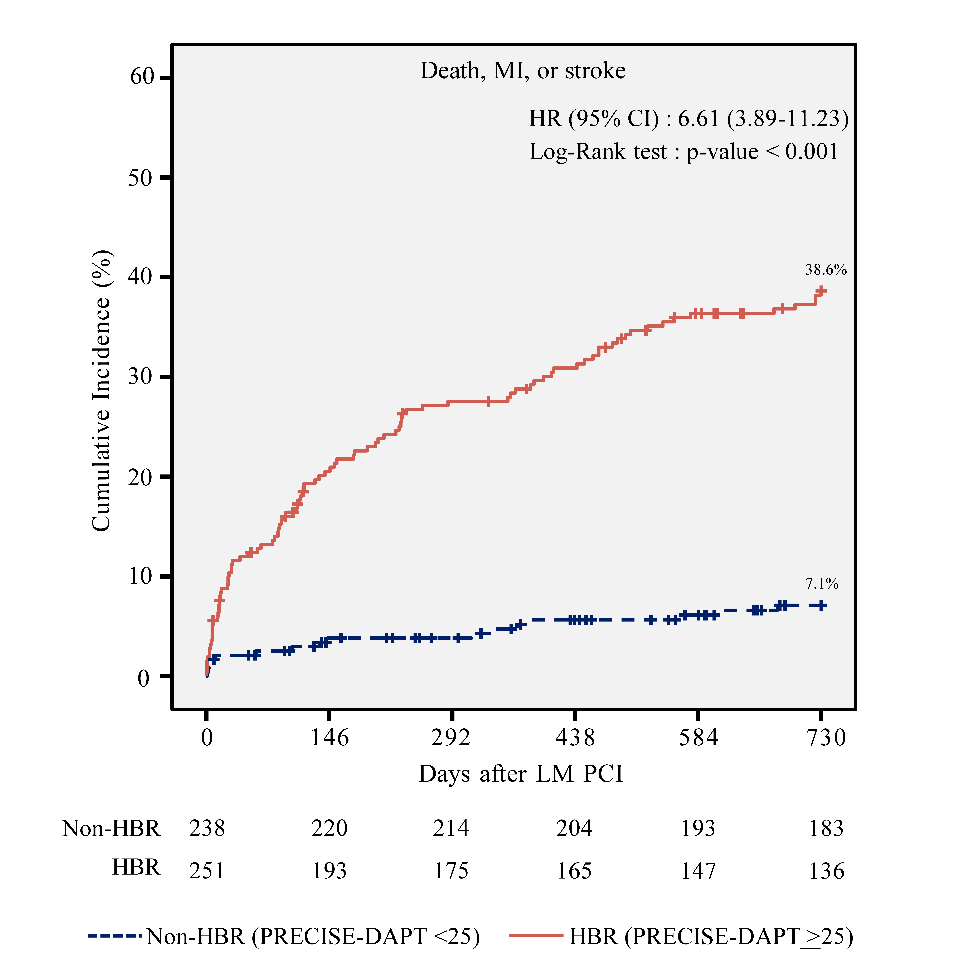


Patients were censored at the last known follow-up date if no event occurred. Censoring was defined using a time scale in days, with 365, 730, and 1825 days corresponding to 1, 2, and 5 years, respectively.

Censoring occurred in 5.5% (27/489) of patients by 1 year, 12.3% (60/489) by 2 years, and 33.5% (164/489) by 5 years.

**Supplementary Figure S4. Cumulative two-year incidence of bleeding events**


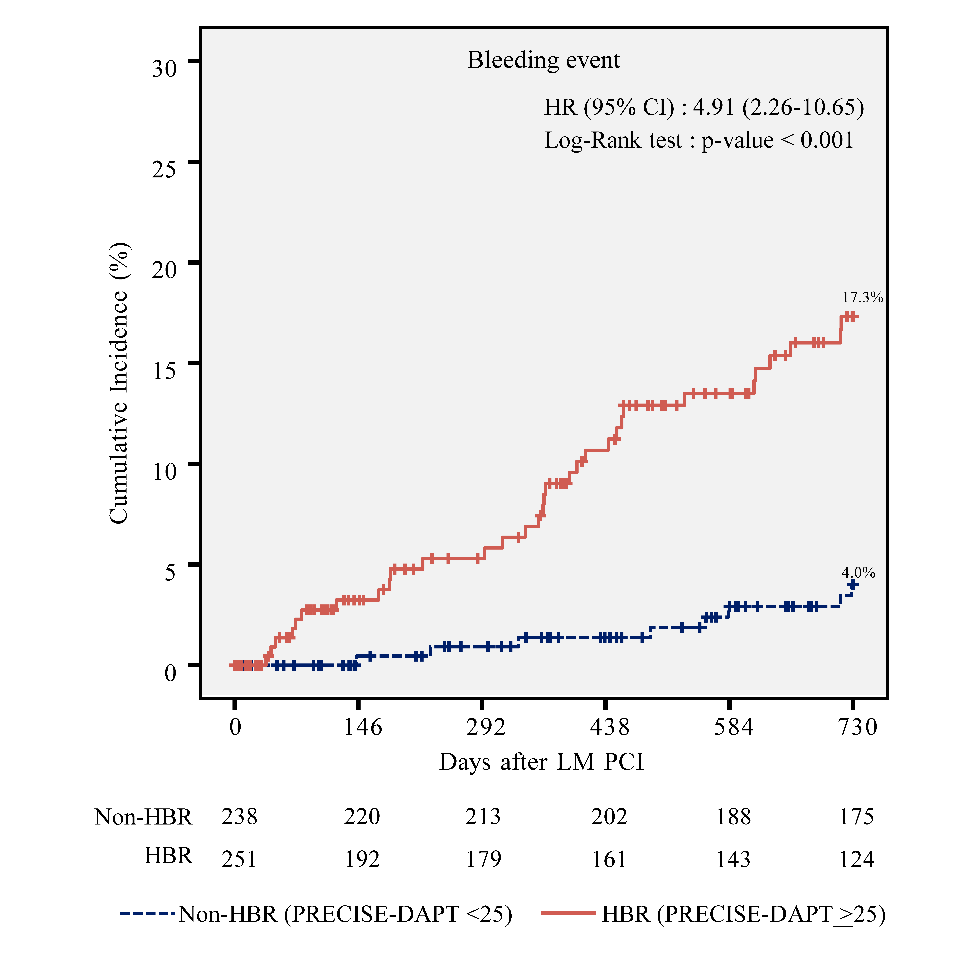


For the secondary outcome of bleeding, censoring occurred in 18.6% (91/489) of patients by 1 year, 30.7% (150/489) by 2 years, and 61.6% (301/489) by 5 years. Given the substantial proportion of patients were censored, especially beyond 2 years, which may affect the stability of long-term estimates and the result beyond this timepoint should be interpreted with caution.

**Supplementary Figure S5. Cumulative five-year incidence of major adverse cardiovascular and cerebrovascular events**


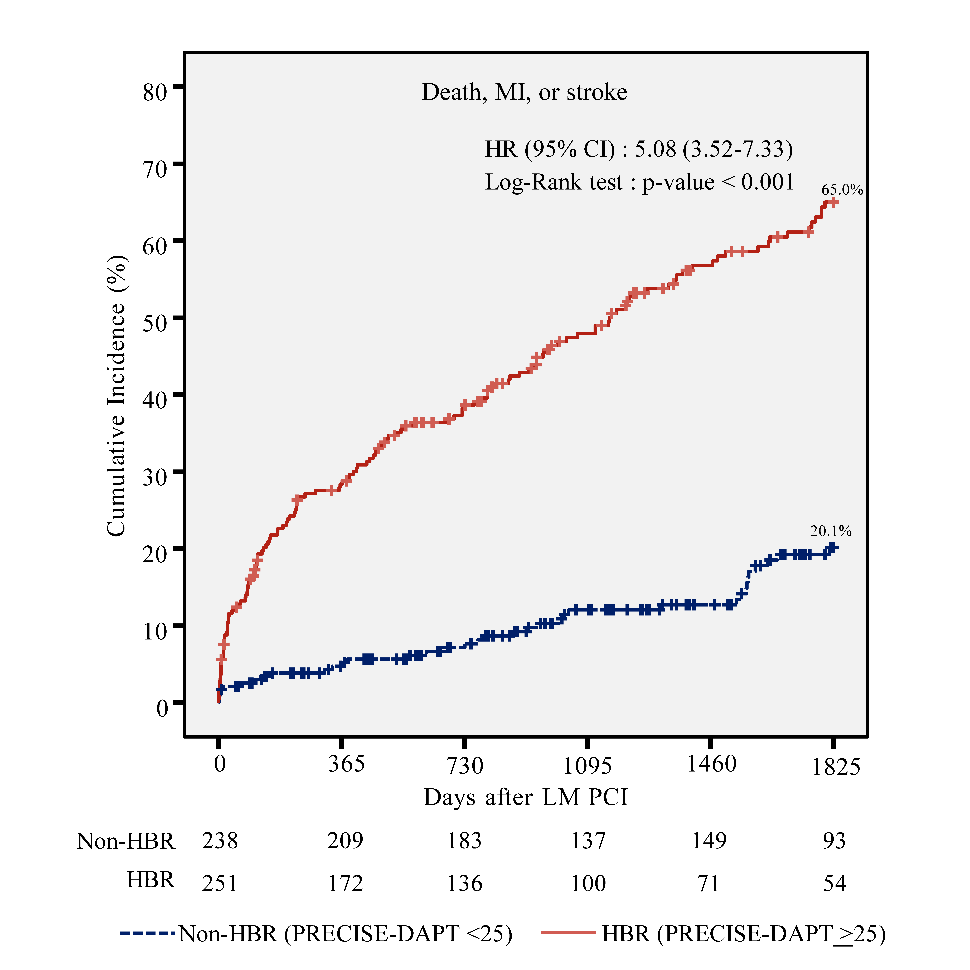


Patients were censored at the last known follow-up date if no event occurred. Censoring was defined using a time scale in days, with 365, 730, and 1825 days corresponding to 1, 2, and 5 years, respectively.

Censoring occurred in 5.5% (27/489) of patients by 1 year, 12.3% (60/489) by 2 years, and 33.5% (164/489) by 5 years.

**Supplementary Figure S6. Cumulative five-year incidence of bleeding events**


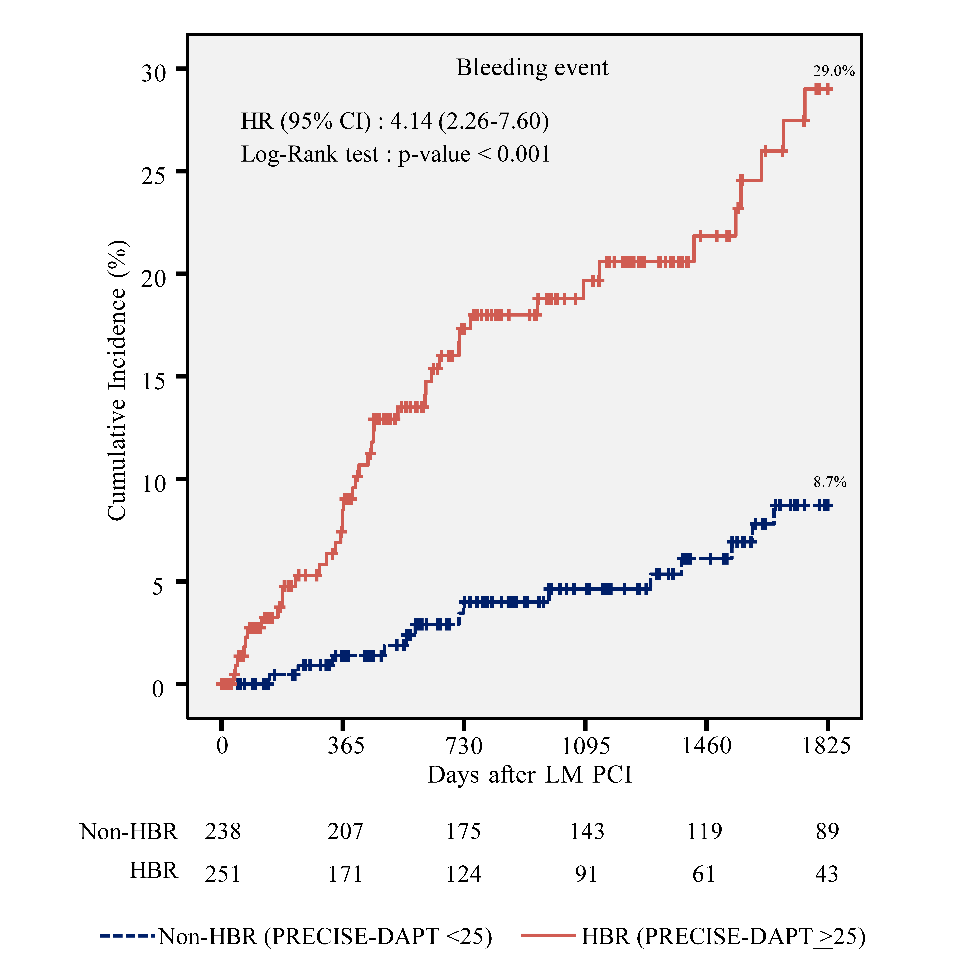


For the secondary outcome of bleeding, censoring occurred in 18.6% (91/489) of patients by 1 year, 30.7% (150/489) by 2 years, and 61.6% (301/489) by 5 years. Given the substantial proportion of patients were censored, especially beyond 2 years, which may affect the stability of long-term estimates and the result beyond this timepoint should be interpreted with caution.
